# Consistency evaluation protocol: A reproducible framework for assessing large language model output repeatability

**DOI:** 10.1016/j.mex.2026.104069

**Published:** 2026-07-25

**Authors:** Shraddha Vaidya, Jatinderkumar R. Saini

**Affiliations:** Symbiosis Institute of Computer Studies and Research, Symbiosis International (Deemed University), Pune, India

**Keywords:** Large language models (LLMs), Output repeatability, Semantic consistency, Structural variability, Composite consistency score (CCS), Reproducible evaluation protocol, Prompt sensitivity, Reliability assessment

## Abstract

The stochastic behaviour of Large Language Models (LLMs) generates varied responses when prompted with the same inputs and parameters. Recent researchers have explored various techniques to understand this behaviour of LLMs, including uncertainty measures, semantic consistency, robustness, and prompt sensitivity. However, the research lacks a reproducible methodology that combines semantic and structural parameters. Thus, the proposed consistency evaluation protocol focuses on evaluating the consistency of LLM responses through repeated prompting, extracting relevant features, and analysing structural and semantic stability through consistency scores. The framework was evaluated using Mistral-7B-Instruct-v0.2 on a set of 20 input texts. For each input text, five independent responses were generated. For each of the generated responses, semantic consistency is determined through embedding-based text representations. In addition, the structural variation is studied through fluctuations in sentence length, word usage, and lexical diversity. Lastly, these measurements are combined to form the Composite Consistency Score (CCS). Further, the validation tests are performed, containing temperature sensitivity, prompt sensitivity, and metric validations reflecting reliable and reproducible consistency evaluations.

## Highlights of this protocol are as follows

• A reproducible protocol is developed for quantifying LLM output repeatability through repeated prompt execution and multiple generations.
• A Composite Consistency Score (CCS) is proposed, which combines semantic similarity and structural variability in the LLM responses.
• A reliable and reproducible validation was performed based on temperature sensitivity, prompt sensitivity, and metric validation that support the consistency evaluation across LLMs.

## Graphical abstract

Graphical abstract of the proposed Consistency Evaluation Protocol.

**Figure:**
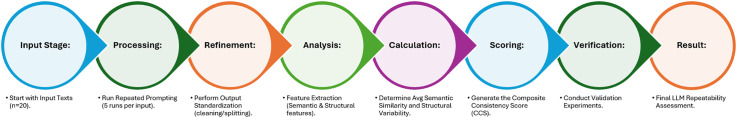

## Specifications table

**Table:** 

Subject area	Computer Science
More specific subject area	Artificial Intelligence, Natural Language Processing, Large Language Model Evaluation
Name of your method	Consistency Evaluation Protocol (CEP) for Assessing Repeatability in Large Language Model Outputs
Name and reference of the original method	No direct original method. The protocol combines repeated prompting, embedding-based semantic similarity analysis, structural variability assessment, and Composite Consistency Score (CCS) computation into a unified evaluation framework.
Resource availability	Kaggle Notebook implementation: Kaggle Implementation Repository

## Background

Recent advances in generative artificial intelligence have led to the development of powerful Large Language Models (LLMs). Although LLMs are capable of producing fluent and contextually relevant responses, they are not deterministic [1,2]. The LLMs make predictions with probability-driven approaches, along with factors such as temperature and sampling methods. Therefore, the same input can produce different responses when executed multiple times. The difference may lie in lexical, structural, and response length, or, to some extent, may affect the underlying meaning of the output [3].

This variability in the output by LLMs affects the trustworthiness of the model and its usage in the real world. The researchers rely on the baseline assumptions about the comparable responses by the LLMs under the same conditions for repeatable executions [4]. However, recent research focuses predominantly on emphasizing performance-oriented measures such as accuracy, F1-score, BLEU, etc. Nevertheless, less attention is paid to the repeatability of generated outputs, and it is insufficiently measured [3].

Researchers have studied the behavior of LLMs across repeated generations and the reliability of their generated responses. The self-consistency-based analyses focus on creating multiple responses for the same prompt. They further analyzed the responses to improve the final answer [5]. ProSA and POSIX, the prompt sensitivity frameworks, observe the responses of LLMs under semantically equivalent prompt reformulations. Another line of research focuses on detecting the robustness of the LLMs with perturbed inputs and multiple repetitions. For example, SCORE and RELIABLEEVAL are frameworks that measure the extent to which model behaviour remains stable across diverse conditions [6,7]. Uncertainty-based assessments in LLMs focus on entropy-based metrics, confidence computation, and probabilistic evaluations. They aim at capturing variability in model behaviour. These methods depict that the LLM responses may differ when executed multiple times, even though with the same inputs and prompts [8,9]. Therefore, the reliability evaluation of LLMs across repeated generations has become a significant area of investigation.

Another direction in this research focuses on the semantic consistency and structural characteristics evaluation of LLM responses. For semantic consistency evaluation, recent techniques such as BERTScore were adopted, which capture the semantic relationship among text embeddings [10]. Another set of methods, including AXCEL, HCSEM, and SMILE, incorporates semantic, lexical, and syntactic information to analyse whether the LLM responses match closely with human perceptions of text quality [[11], [12], [13]]. The StructEval approach shows that semantic correctness alone is not sufficient to fully capture the quality of the response. StructEval emphasizes that the structural characteristics are essential in evaluating the consistency and repeatability of the responses generated [14]. Thus, consistency of response is a multi-faceted concept. Therefore, a reliable and trustworthy LLM response must consider both semantic robustness and structural uniformity in the outputs generated by LLMs. Table A from the Appendix section shows the comparison of various approaches and the need for the proposed protocol.

Although researchers have put in efforts to understand the reliability and consistency of LLM responses, they mostly focus on uncertainty, robustness, prompt sensitivity, and semantic quality. In addition, their research mainly focuses on tailored evaluation methodologies and benchmark tasks. Thus, a practical and reproducible method is required that can evaluate both the semantic consistency and structural stability. Therefore, in this research, the authors propose a protocol to evaluate the repeatability of LLM-generated responses.

The protocol generates multiple responses from a fixed input dataset of research paper abstracts and computes semantic similarity and structural variability using relevant features. A composite consistency score is generated that captures both dimensions of semantic consistency and structural variability in a single measure. Further, controlled experiments are carried out to investigate the influence of generation parameters and prompt design. In addition, validation checks are performed for the proposed evaluation metrics. Therefore, the protocol provides a generalizable workflow for consistency checking, which can be applied across LLM architectures.

## Description of protocol

**Fig. 1:**
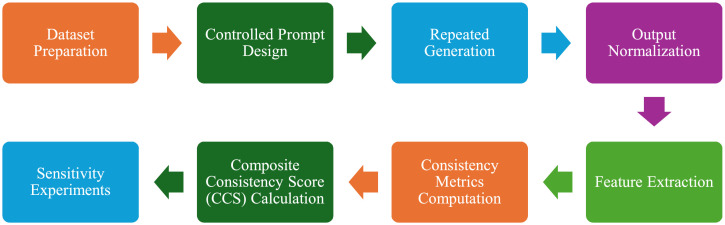
Proposed protocol framework.

This section elaborates on the complete methodology of the proposed consistency evaluation protocol. The protocol aims at quantifying the stability of responses generated by LLMs when the same input and prompts are executed multiple times. The two dimensions of consistency are considered in this protocol: semantic consistency and structural consistency. The semantic consistency captures whether the same meaning is preserved across generations, while structural consistency measures the format of the output generated by LLMs. There are eight phases in this protocol, as depicted in Fig. 1. Algorithm 1 summarizes the complete protocol.

**Algorithm 1:** Protocol for evaluating LLM consistency and repeatability.

Input: • Fixed input corpus (D = {x_1, x_2, …, x_n}). • Prompt template (P). • LLM generation model (M). • Embedding model (E). • Number of repeated runs (R). • Generation parameters. • Structural penalty weight (λ). Output: • Semantic consistency scores. • Structural variability scores. • Composite Consistency Score. Procedure: 1. Set the fixed input dataset (D). 2. Assign a unique identifier for each input text in D. 3. Insert each input text into the fixed prompt template. 4. For each input text, generate (R) independent outputs using identical generation parameters. 5. Store all raw outputs with text identifiers and run identifiers. 6. Clean each generated output by removing prefixes, numbering, bullet points, and extra whitespace. 7. Segment each cleaned output into sentences. 8. Extract sentence count, word count, and lexical diversity. 9. Encode all outputs using the embedding model. 10. Compute pairwise cosine similarity among repeated outputs for each input. 11. Calculate average semantic similarity for each input. 12. Compute sentence-count standard deviation and word-count standard deviation. 13. Normalize word-count variability. 14. Compute Composite Consistency Score.

### Dataset preparation

The dataset consists of 20 research paper abstracts picked from the Scopus database. The abstracts belong to multiple domains, including AI, computer vision, healthcare, deepfakes, IoT, and quantum computing. Thus, making the protocol capable of assessing the applicability in various domains. The 20 abstracts were called input text and were limited to 120–125 words each. This is essential as it remains informative as required for summarization, and it is within the range of the LLM token limit for quick and efficient processing. Each input text is numbered from T01 to T20, and the text was not modified throughout the experimentation.

### Controlled prompt design

A strict prompt was designed with two main purposes: first, it must reduce ambiguity in the generation task, and secondly, it will assist in measuring structural consistency. The strict prompt consisted of the instruction “Summarize the following text into EXACTLY 3 sentences. Output should not contain bullet points, numbering, extra formatting”. This helps in removing variations in output length and structure from task ambiguity. The same prompt was used for all the texts in the main experiment without any changes.

### Repeated independent generation

To perform repeated generations, the strict prompt designed in the previous step was executed five times per input text, thereby creating 100 output texts. Every iteration was independent of the other runs. No output from the previous run was passed as an input to the next run, and no chaining or iterative refinement was performed. The text generation settings were as follows and have not been changed across runs or iterations:

• Model: Mistral-7B-Instruct-v0.2.
• Temperature: 0.7.
• Top-p: 1.0.
• Maximum new tokens: 150.
• Runs per input: 5.

The model was executed in a Kaggle notebook environment using GPU acceleration. The repeated generation outputs were saved in a structured CSV format containing the text identifier, run identifier, prompt, generated output, and completion status.

### Output normalization

The outputs generated by the model are normalized to remove prefixes, numbering, quotation marks, and whitespaces, if any. The normalization procedure included the following operations:

1. Removal of common prefixes such as “Summary:” and “Text summary:”.
2. Removal of numbering markers such as “1.”, “2.”, and similar list indicators.
3. Removal of bullet markers such as hyphens, asterisks, or bullet symbols.
4. Removal of unnecessary quotation marks.
5. Normalization of repeated whitespace into a single space.
6. Rule-based sentence segmentation.

Further, punctuation-based boundary detection was used to perform sentence segmentation. Each cleaned output was split into sentences based on full stops, question marks, and exclamation marks. Lastly, for each generated output, the sentence list is computed, which measures the sentence count.

### Feature extraction

At this stage, three structural and lexical features were extracted from every generated output. First is sentence count, which measures the number of sentences in each generated summary. Since the prompt required exactly three sentences, sentence count directly measures structural adherence to the prompt. Secondly, the word count feature measures the total number of words in each generated output. This enables capturing length variability across repeated runs. Even when two outputs contain the same number of sentences, they may differ substantially in length. The third feature computes lexical diversity, calculated as the ratio of unique words to total words. A higher lexical diversity value indicates greater vocabulary variety within an output, while lower values may indicate more repetitive phrasing. These three features provide surface-level structural and lexical signals. Thus, structural consistency is measured through observable output characteristics and not deep syntactic or discourse-level structure.

### Semantic consistency computation

Semantic consistency was measured using sentence embeddings and cosine similarity. Each cleaned output was encoded using the SentenceTransformer model all-MiniLM-L6-v2. This sentence transformer provides lightweight yet effective semantic embeddings for textual similarity analysis. Also, cosine similarity is suitable for semantic comparison as it captures the angular similarity between embeddings rather than absolute magnitude. Thus, embeddings were created for five generated outputs per input text. Further, embeddings of these five outputs were compared pairwise using cosine similarity. These five outputs produce ten unique pairwise comparisons. Therefore, semantic consistency for each input text was computed as the average of these ten cosine similarity scores. For each input, the protocol reports average similarity, minimum similarity, maximum similarity, and similarity standard deviation. A higher average similarity indicates greater semantic repeatability across repeated runs. The semantic consistency score $S_{i}$ for input $x_{i}$ is calculated as in Eq. (1).

(1) $$S_{i}=\frac{2}{R\left(R-1\right)}\sum_{j=1}^{R-1}\sum_{k=j+1}^{R}cos\left(eij,\;eik\right)$$

The value of $S_{i}$ lies between 0 and 1. $S_{i}\approx 1\;$indicates very high semantic consistency, meaning repeated outputs preserve nearly identical meaning. $S_{i}\approx 0$ indicates very low semantic similarity, meaning outputs differ substantially in meaning. The symbols used in the equation are explained in Table B of the Appendix section.

### Structural variability computation

The protocol further evaluates structural variability across repeated generations. Structural variability refers to the degree to which the generated outputs differ in format, length, and organization when the same prompt is executed multiple times. Even when two outputs express nearly identical meaning, they may differ in the number of sentences, response length, verbosity, formatting structure, or amount of detail. Therefore, structural variability was measured separately from semantic similarity. The protocol quantifies structural variability using two statistical measures: Sentence-count variability and Word-count variability. Both measures are computed using the standard deviation across repeated outputs.

Thus, Sentence-level structural variability is computed as shown in Eq. (2). The equation computes how much the sentence counts vary across repeated outputs for the same input. Meaning of symbols in the equation is presented in Table C, Appendix section.

(2) $$\sigma_{i}^{sent}=std\left(c_{i1}^{sent},\;c_{i2}^{sent},\;\ldots ,\;c_{iR}^{sent}\right)$$

Low sentence-level variability indicates output contains nearly the same number of sentences, structure is stable, and prompt adherence is strong. High sentence-level variability indicates output differs substantially in structure, prompt adherence is weaker, and generation format is stable. Sentence count alone cannot fully capture structural changes because two outputs may contain the same number of sentences but still differ greatly in length and verbosity. Therefore, the protocol also measures variability in total word count.

Word-level variability measures how much the response lengths vary across repeated generations. It is computed using Eq. (3). The meaning of symbols in Eq. (3) is explained in Table D, the Appendix section. A higher standard deviation indicates varying verbosity, unstable output length, and inconsistent detail level.

(3) $$\sigma_{i}^{word}=std\;\left(c_{i1}^{word},\;c_{i2}^{word},\;\ldots ,\;c_{iR}^{word}\right)$$

Further, word-count variability is normalized as the word-count standard deviation is usually numerically much larger than the sentence-count standard deviation. Eq. (4) rescales all word variability values into a comparable range. Thus, after normalization, values near 0 indicate highly stable output lengths, while values near 1 indicate high variability in output length. This prevents word variability from overpowering sentence variability in the final composite metric. Table E, Appendix, elaborates symbol meanings of Eq. (4).

(4) $${\hat{\sigma }}_{i}^{word}=\frac{\sigma_{i}^{word}}{max\left(\sigma^{word}\right)}$$

### Composite consistency score

The protocol integrates semantic consistency and structural stability into one unified score called the Composite Consistency Score (CCS), Eq. (5). The CCS penalizes outputs that are semantically similar but structurally unstable. In this study, λ, was set to 0.1, a balanced midpoint, since the empirical sensitivity analysis showed stable CSS rankings for λ in [0.05, 0.2]. Thus, λ penalizes structural deviations without overshadowing semantic similarity. A higher CCS indicates stronger overall consistency. A lower CCS indicates that the output may preserve meaning but vary in structure or length. Table F, Appendix, elaborates Eq. (5) variables.

(5) $$CCS_{i}=S_{i}-\lambda \left(\sigma_{i}^{sent}+{\hat{\sigma }}_{i}^{word}\right)$$

## Protocol validation

The protocol validation phases evaluate the measurements of response repeatability by testing its meaning, reproducibility, and interpretability. This section discusses various validation methods adopted in this research.

### Consistency assessment using CCS

This experiment tests whether the protocol quantifies the overall repeatability of LLM outputs by combining semantic and structural consistency. Table 1 displays the summary statistics of the CCS computed over LLM-generated outputs, as described in the methodology section. The mean CCS of 0.82 depicts the moderate-to-high repeatability across evaluated inputs. However, the consistency was not uniform across all the inputs, as evident from the minimum and maximum CCS scores ranging from 0.695 to 0.925. Another observation, some texts produced highly stable summaries, while others showed greater variation in structure and length. Table 2 shows the top five most consistent inputs based on CCS. It can be observed that the sentence standard deviation of zero indicates that the model consistently followed the expected sentence structure across all five runs. The Text_id, T20, showed the highest consistency, supported by high semantic similarity and very low structural variation. In Table 3, Text_id T07 showed the least consistent input, with a CCS score of 0.695. Although its average semantic similarity was still reasonably high (0.890), the score decreased due to high sentence-count variability (1.200) and word-count variability. This demonstrates the usefulness of CCS in revealing structural and length instability. Semantic similarity alone is insufficient for evaluating repeatability, and the proposed CCS provides a more informative consistency measure.

**Table 1:** Summary statistics of composite consistency score.

Metric	Value
Number of input texts	20
Mean CCS	0.822
Standard deviation	0.057
Minimum CCS	0.695
25th percentile	0.809
Median CCS	0.827
75th percentile	0.842
Maximum CCS	0.925

**Table 2:** The top five most consistent inputs based on CCS.

Rank	Text_id	Avg similarity	Sentence std	Word std	Normalized word std	Lexical diversity std	CCS	Consistency label
1	T10	0.933	0.000	1.414	0.083	0.013	0.925	High consistency
2	T11	0.935	0.000	3.763	0.221	0.025	0.913	High consistency
3	T03	0.935	0.000	7.419	0.436	0.031	0.892	High consistency
4	T13	0.886	0.000	5.269	0.309	0.034	0.855	High consistency
5	T01	0.895	0.000	8.718	0.512	0.033	0.844	Moderate consistency

**Table 3:** Bottom five least consistent inputs based on CCS.

Rank	Text_id	Avg. similarity	Sentence std	Word std	Normalized word std	Lexical diversity std	CCS	Consistency label
16	T12	0.932	0.800	9.579	0.562	0.010	0.796	Moderate consistency
17	T02	0.912	0.490	13.749	0.807	0.015	0.782	Moderate consistency
18	T20	0.865	0.400	9.252	0.543	0.040	0.770	Moderate consistency
19	T15	0.918	1.200	16.625	0.976	0.085	0.700	Moderate consistency
20	T07	0.890	1.200	12.759	0.749	0.039	0.695	Low consistency

### Temperature sensitivity analysis

In this experiment, the protocol performance is evaluated for its sensitivity to changes in generation randomness. In LLMs, temperature is considered the decoding parameter that controls the degree of randomness during text generation. Lower temperatures generate deterministic outputs while higher temperatures show increased diversity and variability. The idea of this experiment was to determine whether CCS is able to detect changes in output stability resulting from different temperature settings. To perform temperature sensitivity analyses, one representative text was selected from the benchmark dataset. The input was generated independently five times under each temperature setting (0.3, 0.7, and 1.0), resulting in 15 generated outputs. Lastly, the average pair-wise cosine similarity was computed among repeated outputs at each temperature to find the semantic consistency in the outputs. The sentence count standard deviation was computed to find the structural variability. Table 4 shows the effect of temperature on semantic consistency. It can be seen that the average semantic similarity decreases as the temperature increases from 0.3 to 1.0. This indicates that lower temperature values generate more deterministic outputs, resulting in higher semantic consistency across repeated generations. As the temperature increases, the repeated generations show more randomness and semantic variability.

**Table 4:** Effect of temperature on semantic consistency.

Temperature	Average Similarity	Sentence Std
0.3	0.953	0.4
0.7	0.891	0.0
1.0	0.852	0.0

### Prompt sensitivity analysis

The objective of this experiment is to determine whether CCS can quantify the effect of prompt constraints on output repeatability. To determine the semantic consistency differences caused by prompt design, two prompts were designed: strict and loose. The strict prompt imposed a fixed structural constraint: "Summarize the following text into EXACTLY 3 sentences." It enforces output uniformity and minimizes structural variations. The loose prompt: "Summarize the following text." allows the model greater freedom in determining output length and structure. For each prompt type, the same representative input text was generated independently five times using identical generation parameters. The results also demonstrate that both prompt configurations generated high semantic values. This depicts that the models retained the overall meaning of the text irrespective of prompt constraints, as evident from Table 5. The small difference in average similarity indicates that imposing structural restrictions does not substantially affect semantic preservation. A much larger difference was observed in structural behaviour. With loose prompt, the sentence standard deviation was 0.4, and the sentence mean was 5.8, indicating that the model generated outputs with varying numbers of sentences and generally longer summaries. Thus, it is observed that the absence of explicit structural constraints allows greater output variability.

**Table 5:** Prompt sensitivity analysis.

Prompt Type	Average Similarity	Sentence Std	Sentence Mean
Loose	0.969	0.40	5.8
Strict	0.956	0.00	3.0

### Metric Validation

Since the protocol relies on embedding-based cosine similarity as the primary measure of semantic consistency, it is essential to verify that the metric can appropriately differentiate among varying levels of semantic relatedness. To evaluate the performance at three levels: maximum similarity to identical texts, moderate similarity to paraphrased texts, and minimal similarity to unrelated texts. Corresponding three controlled tests are performed: identical text validation, paraphrased text validation, and different text validation. In the first experiment, two identical text passages were compared, which verifies whether the embedding model and cosine similarity computation preserve identity relationships. Table 6 Table A, Table B, Table C, Table D, Table E, Table F depicts the average similarity of 1, showing perfect semantic agreement. Secondly, for paraphrased text validation, two paraphrased passages conveying the same meaning using different vocabulary and sentence structures were compared. A similarity score of 0.713 depicts that the lexical content differed, but the similarity remained substantially higher than that observed for unrelated texts. Lastly, the different text ratios showed an average similarity of 0.065, showing that the similarity metric effectively distinguishes unrelated content.

**Table 6:** Metric validation.

Case	Average Similarity
Identical	1.000
Paraphrased	0.713
Different	0.065

## Limitations and future work

The protocol was validated using a relatively small benchmark dataset consisting of 20 input texts and, therefore, does not capture the full diversity of possible prompting scenarios. The proposed protocol uses only one LLM, while the consistency behaviour may vary across other proprietary and open-source models. In addition, the proposed protocol considers only surface-level output characteristics rather than deeper linguistic properties. Also, semantic consistency was measured using a single embedding model; different embedding models may provide slightly different similarity estimates. The temperature and prompt sensitivity experiments are conducted on a representative input text, which limits the generalizability of those specific observations.

The open areas of research in the future may focus on assessing the factual correctness, detecting hallucinations, or identifying the performance of a specific task. In addition, the proposed protocol can be extended to validate multi-modal data, code generation, reasoning-intensive, or agent-based LLM applications.

## Ethics statement

None.

## CRediT authorship contribution statement

**Shraddha Vaidya:** Conceptualization, Data curation, Validation, Methodology, Software, Writing – original draft. **Jatinderkumar R. Saini:** Validation, Formal analysis.

## Declaration of competing interests

The authors do not have any conflicts of interest.

## Figures and Tables

**Table A:** Comparative analysis of existing methods in LLM consistency evaluation.

Approach	Semantic Evaluation	Structural Evaluation	Repeated Sampling	Reproducible Protocol	Unified Score
SCORE [7]	Partial	No	Yes	Yes	No
ProSA [15]	No	No	Yes	Yes	PromptSensiScore
POSIX [16]	No	No	Yes	Yes	Prompt Sensitivity Index
AXCEL [11]	Yes	Partial	No	No	No
StructEval [14]	Partial	Yes	No	Yes	Structural Metrics
UBENCH [8]	No	No	Yes	Yes	Uncertainty Metrics
RELIABLEEVAL [6]	No	No	Yes	Yes	Reliability Estimator
**Proposed Protocol**	**Yes**	**Yes**	**Yes**	**Yes**	**CCS**

**Table B:** Semantic consistency formula (Eq. (1)).

Symbol	Meaning
$S_{i}$	semantic consistency score for input (x_i)
R	number of repeated runs per input
eij	embedding vector of the (j^{th}) output
eik	embedding vector of the (k^{th}) output
cos⁡(eij,eik)	cosine similarity between two output embeddings
j	index of first output
k	index of the second output

**Table C:** Sentence-level structural variability (Eq. (2)).

Symbol	Meaning
$\sigma_{i}^{\mathrm{sent}}$	sentence-count variability for input $x_{i}$
*Std(.)*	statistical standard deviation function
R	number of repeated runs
$c_{i1}^{\mathrm{sent}}$	sentence count of the $j^{th}$output for input $x_{i}$
i	input text index
j	Repeated run index

**Table D:** Word-level structural variability (Eq. (3)).

Symbol	Meaning
$\sigma_{i}^{\mathrm{word}}$	word-count variability for input $x_{i}$
*Std(.)*	statistical standard deviation function
R	number of repeated runs
$c_{\mathrm{ij}}^{\mathrm{word}}$	word count of the $j^{th}$output for input $x_{i}$
i	input text index
j	Repeated run index

**Table E:** Normalized word variability (Eq. (4)).

Symbol	Meaning
${\hat{\sigma }}_{i}^{\mathrm{word}}$	normalized word variability
$\sigma_{i}^{\mathrm{word}}$	raw word-count variability
$\max(\sigma^{\mathrm{word}})$	maximum observed word variability across all inputs

**Table F:** Composite consistency score (Eq. (5)).

Symbol	Meaning
$\mathrm{CC}S_{i}$	final consistency score for input (x_i)
$S_{i}$	average semantic similarity
$\sigma_{i}^{\mathrm{sent}}$	sentence-level variability
${\hat{\sigma }}_{i}^{\mathrm{word}}$	normalized word-level variability
λ	structural penalty weight

## Data Availability

Data will be made available on request.
